# Precision antimicrobials: the next paradigm shift after broad-spectrum therapy

**DOI:** 10.3389/fmicb.2026.1881800

**Published:** 2026-07-16

**Authors:** Shanmugam N. R. Siva, Sathish Kumar Marimuthu, Manojkumar Kumaran, Nagendran Tharmalingam

**Affiliations:** 1 Department of Food Science and Technology, Nebraska Food for Health Center, University of Nebraska – Lincoln, Lincoln, NE, USA; 2 Kirnd Institute of Research & Development, Tiruchirappalli, Tamil Nadu, India; 3 Lab of Axon Growth and Development, Council of Scientific and Industrial Research (CSIR) – Center for Cellular and Molecular Biology (CCMB), Hyderabad, Telangana, India; 4 Houston Methodist Academic Institute, Houston, TX, United States; 5 Weill Cornell Medical College, Cornell University, New York, NY, United States

**Keywords:** antimicrobial resistance, artificial intelligence-driven drug discovery, broad-spectrum antibiotics, multi-omics, precision antimicrobials

## 1 Introduction

Antimicrobial therapy, developed over the past century, has historically relied on broad-spectrum agents that are effective against a wide range of bacterial pathogens (Muteeb et al., 2023). Although the approach restored global health, the rapid rise in antimicrobial resistance (AMR) and the shift in commensal microbiota show the shortcomings of treating diverse pathogens with uniform chemical pressures (Huang et al., 2025). Each dose of a broad-spectrum antibiotic reshapes microbial communities, disrupts host-microbiome interactions, and exerts selective pressure that accelerates the emergence and spread of resistance genes (Li et al., 2025). As AMR rates increase across major clinical pathogens, the medical community is confronting a stark reality: what once worked reliably can no longer be assumed safe, sustainable, or effective (Ahmed et al., 2024).

Precision antimicrobials are an alternative to broad-spectrum antibiotics. Precision antimicrobials act in two ways: either they directly kill the pathogenic organism with minimal side effects, or they specifically inhibit a crucial node in pathogenesis, thereby interfering with the pathogen's maintenance and/or persistence in the host (Paharik et al., 2017). Precision antimicrobial agents that selectively target specific pathogens, molecular pathways, or resistance mechanisms offer a tailored strategy to combat AMR (de la Fuente-Nunez et al., 2017). Advances in whole-genome sequencing, systems biology, and laboratory automation reveal a pathogen diversity at unprecedented resolution, showing that strains within the same species can differ dramatically in virulence, resistance determinants, and metabolic dependencies (Tedersoo et al., 2019). However, new computational tools enable rapid interpretation and linking genomic signatures to functional vulnerabilities (Clark and Lillard Jr, 2024). Precision therapeutics aims to design pathogen-specific, mechanism-aware, and adaptable antimicrobial interventions that are responsive to evolving resistance landscapes (Spaulding et al., 2018).

Such transition shifts are seen in both oncology and immunology, where personalized therapy has superseded one-size-fits-all approaches (Tomasello et al., 2026). While precision antimicrobial strategies borrow conceptual inspiration from oncology precision medicine, infectious disease care is fundamentally different because infections may be polymicrobial, pathogens can evolve rapidly, and many patients require immediate empiric therapy before diagnostic results are available (Bissonnette and Bergeron, 2012). Accordingly, precision antimicrobials should be presented as a complement to, rather than a replacement for, timely empiric treatment in acute care settings. However, realizing this vision requires more than biological insight. It demands computational/algorithmic innovations that are capable of predicting effective targets, identifying optimal compounds, as well as navigating the complexity of host-pathogen interactions (Wong et al., 2023).

**Figure 1:**
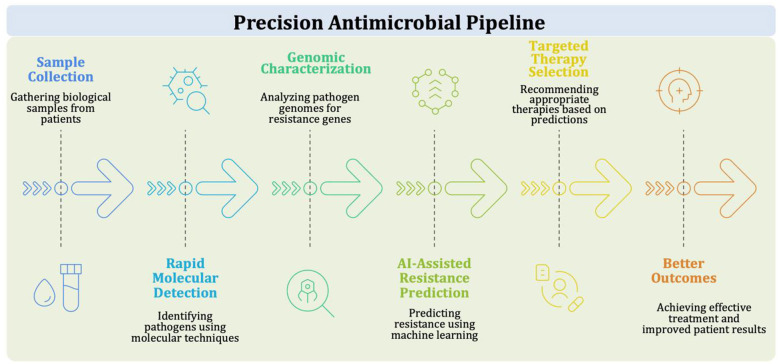
A schematic figure illustrating the precision antimicrobial pipeline.

This opinion article outlines how precision antimicrobials can reshape the landscape of therapeutic development (Figure 1). We first describe the limitations of broad-spectrum therapy, then explore how multi-omics data can pinpoint actionable molecular targets and discuss the centerpiece of this transition: the rise of artificial intelligence (AI) and computational modeling as engines for rational antimicrobial design. Finally, we highlight how digital predictions inform clinical decision-making, forming an iterative pipeline that links molecular data to actionable therapies. Together, these components represent a transformative framework for the next-generation precision antimicrobial discovery.

## 2 The limits of broad-spectrum therapy

Broad-spectrum antibiotics provide empirical coverage against a wide range of Gram-positive and Gram-negative bacteria, including AMR strains, while awaiting culture results. They are particularly important in settings without rapid diagnostics and in intensive care units (ICUs), where delays in antimicrobial therapy are associated with increased mortality (Rhee et al., 2020; Timsit et al., 2024). Despite these advantages, indiscriminate reliance on broad-spectrum coverage is increasingly being questioned because it can drive resistance selection and microbiome disruption even when narrower options would have sufficed. However, this critique should be tempered by the fact that broad-spectrum therapy remains essential in septic shock, ICU empiric treatment, polymicrobial infections, and settings of substantial diagnostic uncertainty, where delayed or overly narrow therapy can worsen outcomes (Strich et al., 2020).

### 2.1 Adverse effects of broad-spectrum antibiotic use

A study by Webb et al. involving 1,995 patients with community-acquired pneumonia reported that 39.7% received broad-spectrum antibiotics, whereas only 3% of cases were caused by multidrug-resistant (MDR) bacteria, highlighting the potential overuse of broad empirical antimicrobial therapy (Webb et al., 2019). After adjusting for confounders, broad-spectrum antibiotic use was associated with higher mortality and a 17.5% rate of antibiotic-related adverse events. In cases with negative bacteriological results, alternative diagnoses may have been missed (Webb et al., 2019). In another cohort of 15,183 adults with bacteriologically confirmed community-acquired sepsis, 81.6% received appropriate antibiotics (Rhee et al., 2020). Less than 30% of cases were attributed to MDR pathogens, yet unnecessary broad-spectrum antibiotics were administered in 67.8% of patients. This inappropriate use was linked to increased hospital mortality (adjusted OR 1.27, 95% CI 1.06–1.40) and higher rates of acute kidney injury and *Clostridioides difficile* infections. Furthermore, studies on nosocomial infections suggest that dual antibiotic therapy offers no significant advantage over single-agent treatment while increasing overall antibiotic exposure (Barbier et al., 2024).

### 2.2 Microbiome disruption associated with antibiotic use

One major disadvantage of broad-spectrum antibiotics is their disruption of the microbiome. By non-selectively eliminating both pathogenic and beneficial bacteria, broad-spectrum agents can cause dysbiosis, increasing the risk of opportunistic infections such as *C. difficile* colitis (Dahiya and Nigam, 2023). Dysbiosis impairs the balance of the gut microbial ecosystem, reducing its intrinsic resistance and protective capacity (Levy et al., 2017). Changes to gut microbiota are increasingly implicated to several non-communicable diseases. Persistent dysbiosis may drive the progression of manageable conditions into chronic disorders and has been associated with gastrointestinal diseases, liver disorders, colorectal cancer, diabetes, and dermatological conditions such as eczema. Furthermore, antibiotic-induced dysbiosis can lead to diarrhea and recurrent infections caused by *C. difficile* (Duvallet et al., 2017; Wirbel et al., 2019). A 2025 study using 16S rRNA gene sequencing examined the short- and long-term effects of commonly used antibiotics ampicillin, vancomycin, metronidazole, and neomycin on murine gut microbiota showed significant antibiotic-induced alterations in intestinal microbial composition, leading to gut dysbiosis and competition among bacterial populations (Huang et al., 2025). The study also reported that combination antibiotic therapy, particularly involving quinolones and metronidazole, caused more persistent dysbiosis than treatment with a single antibiotic (Huang et al., 2025).

### 2.3 Diagnostic infrastructure for precision antimicrobial therapy

Precision antimicrobial strategies are only as effective as the diagnostics that guide them. Conventional culture-based identification and susceptibility testing can take 48 to 96 h, a delay incompatible with conditions such as sepsis that demand therapy within the first hour, which is why empiric broad-spectrum treatment remains the default practice (Marin et al., 2025). Rapid multiplex PCR panels (e.g., GeneXpert Carba-R, BioFire) now return pathogen and resistance-gene results within 45 min to a few hours, while culture-independent metagenomic and nanopore sequencing extend this to polymicrobial and culture-negative infections in near real time (Alatawi et al., 2025). However, cost, infrastructure, and trained-personnel requirements still limit access to these tools, and portable nanopore platforms, though cheaper than short-read sequencing, remain constrained in low-resource settings by reagent supply chains and the lack of standardized workflows (Thornval et al., 2025). Closing this diagnostic gap is a prerequisite for precision antimicrobials to move from concept to clinically actionable, globally equitable practice.

## 3 Identifying targets for precision antimicrobials

The precision antimicrobial paradigm anchors drug discovery to critical molecular targets of the bacterial pathogen, structurally absent or sufficiently divergent in the human host, and accessible to pharmacological intervention (Lewis, 2013; Tommasi et al., 2015). The strategy demands a systematic framework integrating genomic essentiality data, structural biology, and translational feasibility into a coherent prioritization logic (Brown and Wright, 2016; Tommasi et al., 2015). The first non-negotiable criterion is essentiality: gene loss or protein inhibition must be a critical target that **impairs** bacterial viability in the host (Payne et al., 2007). Genome-wide screens using transposon insertion sequencing (Tn-seq) and CRISPRi have systematically arranged essential genes across priority pathogens including *Staphylococcus aureus, Mycobacterium tuberculosis, Klebsiella pneumoniae, and Acenetobacter baumannii* (Tommasi et al., 2015; Stokes et al., 2020). Essentiality must be paired with selectivity, exploiting fundamental prokaryotic-eukaryotic discontinuities: 70S vs. 80S ribosome architecture, type II vs. type I fatty acid synthase organization, and the absence of two-component signal transduction in mammalian physiology (Brown and Wright, 2016; Farha and Brown, 2019). These intrinsic differences define selectivity windows through which pathogen-targeted agents operate with minimal host toxicity (Payne et al., 2007; Tommasi et al., 2015).

Table 1 integrates twelve priority target categories organized into four functional groups (cell envelope, information processing, regulatory/adaptive, and proteostasis/resistance modulators) alongside their selectivity basis, key mechanistic advances, and the validation technologies enabling their identification. Rapid advances in structural modeling from AlphaFold2, chemoproteomics (including activity-based protein profiling (ABPP) and thermal proteome profiling (TPP)), and pan-genomic studies on clinical isolate collections have fundamentally altered the pace of target discovery, revealing polymorphisms that distinguish patient populations by predicted inhibitor susceptibility, a diagnostic approach similar to oncology biomarker projects (Boucher et al., 2009; Stokes et al., 2020; Tommasi et al., 2015).

**Table 1:** Precision antimicrobial targets: molecular components, representative agents, selectivity basis, and validation technologies, organized by functional group.

Target category	Biological pathway	Key molecular components	Representative agents	Organism spectrum	Selectivity basis & mechanistic highlights	Validation technologies
*A. Cell envelope targets*						
Peptidoglycan synthesis	Cell wall biosynthesis	PBPs, MurA–MurF, MraY, FtsZ	Beta-lactams, Fosfomycin, Ramoplanin, Teixobactin	ESKAPE pathogens; Gram-positives	Pathway absent in eukaryotes; FtsZ (prokaryotic tubulin, no mammalian counterpart): anti-MRSA compounds PC190723, TXA-709; Teixobactin: resistance-refractory lipid II/III binding bypassing glycopeptide resistance	Tn-seq essentiality profiling; Cryo-EM of PBP complexes; iChip cultivation platform
Type II fatty acid synthesis (FASII)	Lipid membrane assembly	FabI, FabB, FabF, FabH, FabG	Triclosan, Platensimycin, Afabicin (Debio1452)	S. aureus, H. pylori, M. *tuberculosis*	Type II FAS (discrete monofunctional enzymes) vs. mammalian type I (single multifunctional polypeptide); Afabicin: staphylococcal FabI prodrug, clinical approval for bone/joint infections	CRISPRi conditional essentiality; ABPP target engagement
LPS biosynthesis	Outer membrane integrity	LpxC, LpxA, LpxD, MsbA transporter	CHIR-090, LpxC inhibitors	Gram-negatives (Acinetobacter, P. *aeruginosa*)	Lipid A–based outer leaflet unique to Gram-negatives, absent in eukaryotes; LpxC catalyzes committed step in lipid A synthesis	AlphaFold2 structure prediction; Virtual screening
15.6-7.4,-25.3690ptBacterial cytoplasmic membrane	Membrane potential & ion homeostasis	Lipid II flippase, Cardiolipin synthase, MprF	Daptomycin, Polymyxins, Teixobactin	VRE, MRSA, MDR Gram-negatives	Membrane disruption achieves clinically relevant selectivity against resistant Gram-positive and Gram-negative organisms	TPP selectivity profiling
*B. Information processing targets*						
DNA Gyrase & Topoisomerase IV	DNA replication & supercoiling	GyrA, GyrB, ParC, ParE	Fluoroquinolones, Aminocoumarins, Zoliflodacin	Broad-spectrum; evolving resistance variants	Bacterial GyrA/GyrB introduces negative supercoils; differs structurally from eukaryotic Topo II; Zoliflodacin: non-fluoroquinolone binding mode for N. gonorrhoeae	Pan-genomic isolate analysis; Companion diagnostic logic
RNA Polymerase (RNAP)	Transcription initiation & elongation	RpoB, RpoC, sigma factors	Rifamycins, Fidaxomicin, Lipiarmycin	M. tuberculosis, C. *difficile*	Bacterial RNAP (α_2_ββ'ω + sigma) distinct from eukaryotic Pol I/II/III; Fidaxomicin: switch-region inhibitor with spectrum restricted to C. difficile	Cryo-EM sub-site mapping; Structural genomics
15.6-7.4,-38.3690ptBacterial Ribosomes (70S)	Protein translation	16S rRNA, 23S rRNA, L3, L4, S12	Macrolides, Aminoglycosides, Oxazolidinones, Plazomicin	Resistant TB, Gram-negatives	70S (30S + 50S) vs. eukaryotic 80S (40S+60S); Cryo-EM reveals species-restricted helix 69 and L4/L22 tunnel loops for taxonomically selective inhibitor design	Cryo-EM structural biology; AlphaFold2 modeling
*C. Regulatory & adaptive targets*						
Two-Component Signal Transduction	Adaptive stress response & virulence	WalRK, PhoQP, VncRS, YhcSR	Walkmycin, Signermycin B, Closantel	S. aureus, *Streptococcus*, E. coli	TCS (histidine kinase + response regulator) is completely absent in mammals; >30 TCS in S. aureus; WalRK is essential for peptidoglycan homeostasis	Tn-seq; CRISPRi conditional knockdown
Biofilm Regulatory Networks	Biofilm formation & persistence	LuxS/AI-2, PelB, PslA, BioA	Carolacton, Savirin, Furanone C-30 derivatives	P. *aeruginosa*, S. *epidermidis*	Anti-virulence via quorum sensing inhibition (Agr, LuxS/AI-2): disarms pathogenicity without lethal selection pressure, reducing resistance evolution	Metagenomics; ABPP
*D. Proteostasis & resistance modulators*						
Clp Protease System	Protein homeostasis & persistence	ClpP, ClpX, ClpC1, ClpB	Acyldepsipeptides (ADEPs), Bortezomib analogs	M. tuberculosis, S. *aureus*	ADEPs dysregulate ClpP into uncontrolled protease → FtsZ degradation → kills persisters refractory to conventional bactericidal mechanisms; Cyclomarin A: ClpC1 inhibitor for intramacrophage M. tuberculosis	ABPP; TPP; Cryo-EM
Efflux Pump Systems	Antibiotic extrusion & MDR	MexAB-OprM, AcrAB-TolC, NorA, MdfA	PAβN, NMP, D13-9001 (EPIs)	MDR Gram-negatives, MRSA	RND-family pumps are constitutive membrane components; overexpression = most common initial MDR step; EPIs restore multi-class antibiotic susceptibility simultaneously	Pan-genomic resistance genotyping
Folate Biosynthesis Pathway	Nucleotide precursor synthesis	DHPS (FolP), DHFR (FolA)	Sulfonamides, Trimethoprim, Iclaprim	Pneumocystis, Nocardia, MRSA	Folate synthesis essential in bacteria but absent in mammals (humans obtain folate from diet)	AlphaFold2; Virtual screening

PBP, penicillin-binding protein; FASII, type II fatty acid synthesis; TCS, two-component signal system; MDR, multidrug-resistant; MRSA, methicillin-resistant S. aureus; VRE, vancomycin-resistant enterococci; EPI, efflux pump inhibitor; ESKAPE, Enterococcus faecium, S. aureus, K. pneumoniae, A. baumannii, P. aeruginosa, Enterobacter spp.; ABPP, activity-based protein profiling; TPP, thermal proteome profiling.

The four functional groups in Table 1 are arranged to reflect a hierarchy of selectivity, moving from targets that are structurally absent from human cells to those that are merely divergent in sequence or regulation. Group A (cell envelope) and Group B (information processing) targets offer the cleanest selectivity windows, since structures such as FtsZ, the bacterial 70S ribosome, and the type II fatty acid synthase system have no direct human counterpart, which is why several agents derived from this group, including teixobactin, fidaxomicin, and afabicin, have reached clinical evaluation. Group C (regulatory and adaptive) targets such as two-component signal transduction systems are attractive precisely because they are entirely absent in mammalian physiology, but most inhibitors remain at an earlier discovery stage because these systems are less biochemically tractable than classical enzyme targets. Group D (proteostasis and resistance modulators) illustrates a complementary strategy: rather than killing bacteria outright, agents such as efflux pump inhibitors and ClpP dysregulators restore the efficacy of existing antibiotics or eliminate persister cells that evade conventional bactericidal mechanisms. Read together, the table indicates that the validation technologies are not interchangeable, but cumulative: Tn-seq and CRISPRi typically establish essentiality first, structural methods such as cryo-EM and AlphaFold2 then resolve the selectivity basis at atomic resolution, and chemoproteomic platforms (ABPP, TPP) subsequently confirm target engagement in cells, together forming the prioritization pipeline that determines which targets are mature enough for medicinal chemistry investment.

## 4 AI and computational strategies for drug design

Artificial intelligence is increasingly useful for prioritizing targets, assessing candidate compounds, and integrating genomic, phenotypic, and structural data in antimicrobial research (Zavaleta-Monestel et al., 2025). However, its output must be interpreted cautiously because model performance can be limited by training-set bias, sparse representation of certain pathogens, uneven annotation quality, and weak external validation across laboratories, species, and clinical settings (Serrano et al., 2024; Sakagianni et al., 2024). In addition, AI-generated structure-function relationships may appear plausible but remain unverified, and some predictions can fail when transferred to new datasets or real biological systems. These limitations mean that AI should be treated as a decision-support tool that helps generate hypotheses, rather than as a substitute for experimental validation (Aithani et al., 2023; Bakhtiyar et al., 2025; Mamoudou and Mune, 2025).

Machine learning models trained on genomic and phenotypic data can now infer pathogen vulnerabilities directly from mutational signatures or resistance trajectories (Hossain and Yousefi, 2026). Neural networks integrate comparative genomics, essential gene datasets, and multi-omics profiles to identify promising targets that differentiate between strains or species, to develop antibiotics that do not affect beneficial bacteria in the environment (Przymus et al., 2024; Wang et al., 2025). Predictive modeling also enables researchers to map resistance pathways before they emerge experimentally, facilitating the selection of compounds that are less prone to rapid resistance evolution (Jacobs et al., 2024). The use of such technologies is consistent with recent developments that indicate that it is possible to predict mechanism of action (MoA) with the help of deep learning solely based on variant signatures (Krentzel et al., 2026).

Rational drug design has been further revolutionized by advances in structural prediction and computational modeling (Mozaffari et al., 2025). Tools such as Alpha Fold-derived structural models, physics-informed scoring functions, and generative molecular design frameworks enable the rapid prediction of ligand–target interactions (Sim et al., 2025). Large-scale parallel docking and simulation studies conducted on GPU-based systems virtually test millions of potential drugs, identifying only high-affinity compounds while discarding those that might cause adverse side effects due to interactions with non-targeted sites (Blanes-Mira et al., 2022). The application of reinforcement learning methods to optimize lead molecules considers toxicity and specificity constraints (Gow et al., 2022).

Network-based computational strategies add another dimension by integrating host–pathogen interactions, metabolic dependencies, and regulatory circuitry (Durmuş et al., 2015). The approach allows for the creation of targeted antimicrobial combinations capable of killing microbes as well as interfering with their adaptation to induce tolerance and persistence (Cantrell et al., 2022). Significantly, AI-enabled drug repurposing can leverage existing drugs against new targets in microbes—a critical factor for AMR-related research (Yakobi and Nwodo, 2025).

Together, these computational approaches establish a foundation for precision antimicrobial development: algorithmic filtering of chemical space, target-specific compound optimization, and predictive modeling of resistance evolution. AI elevates antimicrobial design from trial and error to a rational, data-driven discipline, an essential shift for the post-broad-spectrum era. Considering AI's potential advantages in drug research, there are a number of obstacles and limitations to consider. The availability of appropriate data is one of the main obstacles (Vamathevan et al., 2019). To address the limits of AI-based drug development by treating AI as part of a hybrid pipeline: improve the data, retain people in the loop, and require every prediction to pass experimental and translational validation (Blanco-González et al., 2023).

## 5 From data to clinical application: broad-spectrum antimicrobial activity

The rapid emergence of AMR has accelerated the development of novel broad-spectrum antibacterial agents. BWC0977 is a novel inhibitor of bacterial topoisomerase that targets DNA gyrase and topoisomerase IV, thereby hindering bacterial DNA synthesis. The drug is extremely effective against multidrug-resistant Gram-positive and Gram-negative bacteria, owing to its low minimum inhibitory concentrations (MICs). Preliminary clinical trial results have suggested the safety and effectiveness of BWC0977 in treating serious infections caused by multidrug-resistant bacteria, such as bacterial pneumonia (Hameed et al., 2024).

The translation of broad-spectrum antibacterial activity into clinical application has also been demonstrated in phase III clinical studies, such as the EAGLE-2 and EAGLE-3 trials, which evaluated the efficacy of the novel antibiotic Gepotidacin compared with Nitrofurantoin for the treatment of uncomplicated urinary tract infections (uUTIs). The findings revealed that Gepotidacin was non-inferior to nitrofurantoin, with superiority in some analyses of EAGLE-3. Overall, the agent had an acceptable tolerability profile, with diarrhea being the most common side effect; however, all adverse effects were mostly mild to moderate, and no severe safety concerns were noted (Wagenlehner et al., 2024).

Antimicrobial peptides (AMPs), along with small-molecule antibacterial agents, are another class of broad-spectrum agents under investigation for therapeutic use. Brilacidin is a synthetic peptide displaying broad-spectrum activity against bacteria, fungi, and viruses, with potent activity against *Staphylococcus aureus*. The compound entered phase II clinical trials as a treatment option for skin infections, coronavirus disease 2019 (COVID-19), and as an oral rinse for mucositis (NCT02052388, NCT04784897, and NCT02324335) (Abdelsattar et al., 2025).

Another promising agent is Exeporfinium chloride, a topical photosensitizing porphyrin derivative with potent activity against Gram-positive bacteria and a low likelihood of resistance development. Developed by Destiny Pharma, the drug completed a phase II clinical trial (NCT03915470) evaluating its ability to reduce nasal colonization of *Staphylococcus aureus* in patients at risk of postoperative infections (Butler et al., 2023).

Similarly, Zosurabalpin inhibits the LptB-FGC complex and blocking lipopolysaccharide export, resulting in cytotoxic levels of lipopolysaccharide retention inside the bacterial cells. In preclinical tests, the drug demonstrated strong antimicrobial activity against carbapenem-resistant *Acinetobacter baumannii*, with selective efficacy and favorable pharmacokinetics. Moreover, phase I clinical trials have shown tolerability and favorable safety data (Terzi et al., 2025).

In parallel with these broad-spectrum candidates, several genuinely precision-guided antibiotics illustrate how the target-prioritization logic described above is already reaching the clinic. Afabicin (Debio 1450), introduced in Table 1 as a FabI inhibitor, is a first-in-class Staphylococcus-selective antibiotic that has advanced through phase II trials for skin and bone/joint infections, including MRSA, while leaving non-staphylococcal gut flora largely intact, since its target enzyme is restricted to staphylococcal fatty acid biosynthesis (Wittke et al., 2020). A complementary, more recently discovered example is fluorofolin, a dihydrofolate reductase inhibitor that exploits a metabolic quirk specific to Pseudomonas aeruginosa, namely its inability to scavenge exogenous thymine, allowing the compound to selectively eliminate this pathogen from mixed-species cultures while sparing co-occurring commensal species (Chain et al., 2024). These agents demonstrate that precision antimicrobials are not purely conceptual: pathogen-restricted essential pathways, once mapped using the genomic and structural approaches outlined in Section 2, can already be translated into selective compounds with demonstrable microbiome-sparing benefits, even though most remain earlier in clinical development than their broad-spectrum counterparts.

Targeted antimicrobial therapies are the key to minimizing AMR and sustaining the microbiome while maintaining clinical effectiveness (Zai et al., 2024). The synergy of multi-omics, functional genomics, and structural biology can generate targets that enable targeted therapeutic interventions for more safer and more effective treatments (Molla and Bitew, 2024). Artificial intelligence and predictive diagnostics facilitate rapid compound selection and the prediction of resistance mechanisms. However, precision antimicrobial therapy depends on rapid and reliable diagnostics that can identify both the pathogen and key resistance determinants within a clinically actionable timeframe (Cesaro et al., 2025; Singh et al., 2026). Biomarker-guided clinical trials, antimicrobial stewardship, and monitoring programs are crucial for the effective use of targeted medicines. Economic and policy measures are required to encourage the development of narrow-spectrum therapies and broad-spectrum medicines as an emergency response (Seok and Park, 2024).

Despite overcoming most aminoglycoside-modifying enzymes, plazomicin remains vulnerable to alternative resistance mechanisms, including efflux pump overexpression, porin alterations, outer-membrane changes, and target-site methylation, highlighting that next-generation antibacterial agents may still face clinically relevant escape pathways that limit long-term efficacy. Similarly, aztreonam/avibactam combines aztreonam's stability to metallo-β-lactamases with avibactam-mediated inhibition of class A, class C, and some class D β-lactamases, supporting activity against many carbapenem-resistant Enterobacterales (Ozdemir et al., 2026).

## 6 Discussion

The use of broad-spectrum antibiotics remains essential for emergencies, but their overuse drives antimicrobial resistance (AMR) and disrupts the microbiome. Therefore, targeted antimicrobial therapies are the key to minimizing AMR while maintaining clinical effectiveness.To develop these therapies, the synergy of multi-omics, functional genomics, and structural biology is necessary to identify specific pathogen targets for safer treatments. Additionally, artificial intelligence helps researchers rapidly select compounds and predict resistance mechanisms before they emerge.

However, precision antimicrobial therapy strongly depends on rapid and reliable diagnostics. These targeted treatments cannot function unless diagnostics identify both the pathogen and its resistance determinants within a clinically actionable timeframe. Finally, to successfully implement this approach, we require biomarker-guided clinical trials, antimicrobial monitoring programs, and policy measures that encourage the development of narrow-spectrum therapies. Precision antimicrobial therapy complements antimicrobial stewardship efforts by facilitating pathogen-directed treatment, whereas stewardship programs provide the clinical framework for ensuring appropriate antimicrobial use. Together, these approaches promote more targeted therapy, reduce unnecessary antimicrobial exposure, and improve treatment efficiency (Watkins, 2022; Shah et al., 2024).

Precision antimicrobial approaches have shown promise in intestinal infections, respiratory infections and inflammatory bowel disease. For instance, oral mannoside therapy reduced ileal colonization by adherent-invasive *Escherichia coli* (AIEC) in a transgenic mouse model of Crohn's disease, supporting the development of the FimH antagonist EB8018 as a targeted therapeutic strategy (Alvarez Dorta et al., 2016). The nasal commensal *Staphylococcus lugdunensis* produces lugdunin, a novel antibiotic that selectively inhibits *Staphylococcus aureus* colonization, highlighting the potential of microbiome-based precision antimicrobial therapies (Zipperer et al., 2016).
